# Occurrence and Levels of Biogenic Amines in Beers Produced by Different Methods

**DOI:** 10.3390/foods10122902

**Published:** 2021-11-23

**Authors:** Katarzyna Nalazek-Rudnicka, Wojciech Wojnowski, Andrzej Wasik

**Affiliations:** Department of Analytical Chemistry, Faculty of Chemistry, Gdańsk University of Technology, 11/12 G. Narutowicza Str., 80-233 Gdańsk, Poland; katnalaz@student.pg.edu.pl (K.N.-R.); wojciech.wojnowski@pg.edu.pl (W.W.)

**Keywords:** biogenic amines, beer, lactic acid bacteria, derivatization, high performance liquid chromatography-tandem mass spectrometry

## Abstract

The concentration of biogenic amines (BAs) in beer depends, among other factors, on the activity of microorganisms, in particular lactic acid bacteria. In this work an analytical method based on derivatization with tosyl chloride and high-performance liquid chromatography-tandem mass spectrometry (HPLC-MS/MS) was used to determine 17 BAs in samples of commercially available beers, and to monitor the changes in concentration of several BAs throughout the fermentation process. In some of the analysed samples the concentration of BAs exceeded the safety threshold for consumers. During the fermentation stage of home-brewing of ale the concentration of spermine in the wort increased until the end of the stormy fermentation, to then drop below the initial concentration at the end of fermentation, and below the LOQ after refermentation. The results of the study indicate that monitoring the total content of BAs is required due to the potential risk to human health.

## 1. Introduction

Beer is a beverage that is generally considered safe with regard to microbiological risk, as the relatively high alcohol content (usually 0.5–10% *w*/*w*), low pH (3.8–4.7), presence of hop-derived iso-α-acids, reduced oxygen level (<0.1 ppm) [1], and other factors do not favour proliferation of most microorganisms [2]. However, certain microorganisms, including in particular the Gram-positive lactic acid bacteria (LAB), can tolerate these adverse conditions and cause the spoilage of beer. It should be noted that certain beer styles, such as Belgian lambics or Berliner Weisse, call for lactic fermentation of the mash with lactic cultures, e.g., *Pedicoccus* or *Lactobacillus delbrueckii* [3]. The activity of LAB, either introduced purposefully or as a result of unwanted contamination, can affect the organoleptic properties of the beverage, but also lead to the formation of biogenic amines (BAs)—low molecular nitrogen compounds. Consumption of foods and drinks in which these compounds are found is generally safe because the human organism is able to metabolize them with the help of monoamine oxidases (MAO) and diaminooxidases (DAO). However, too high a level of BAs in consumed food and beverages may have a detrimental effect on the consumer’s well-being, as the human organism’s detoxification mechanism may not be sufficiently effective. In general, 100 mg/L or 100 mg/kg of BAs is considered as a safe dose for most consumers, but in the case of alcoholic beverages this limit is much lower. This is related to the fact that ethanol may reduce the effectiveness of the detoxification mechanism in humans [4]. In addition, consumers who for some reason take MAO and DAO enzyme inhibitor drugs should also pay attention to the level of BAs in the consumed food and beverages [5]. People who take MAO enzyme inhibitors can suffer from a hypertensive crisis after ingesting certain amounts of tyramine, e.g., by consuming contaminated beer [5,6]. There is also a group of people who, either due to genetics or as a result of gastrointestinal diseases, have insufficiently active DAO enzyme [5].

Based on the literature, the most abundant BAs in beer samples are: agmatine (AGM), histamine (HIS), cadaverine (CAD), putrescine (PUT), 2-phenylethylamine (PHE), tyramine (TYR), spermine (SPE), spermidine (SPD), and histamine (HIS) [7,8,9]. Certain aromatic amines such as TYR and PHE have a vasoconstrictive effect and cause migraine and hypertensive crises, while others such as HIS show vasodilatory action and their ingestion might lead to food poisoning. Some BAs (SPD, SPE, CAD, and PUT) do not affect health, but can form carcinogenic nitrosamines by reacting with nitrites. Furthermore, the concentration level of these BAs could be an indicator of spoilage and quality of food products. Additionally, PUT and CAD can increase the toxicity of other BAs [10,11,12]. Therefore, it is extremely important to monitor the level of BAs in food and alcoholic beverages such as beer. The presence of certain BAs could be used as an indicator of the quality of food products and beverages [13]. Loret et al. proposed the use of a biogenic amines index (BAI) calculated as the ratio of TYR, PUT, CAD, HIS, PHE, and TYR to AGM to determine the microbiological quality of beer fermentation [5]. Lorencová et al. determined eight BAs (HIS, TYR, SPD, SPE, TYR, PHE, PUT and CAD) and their total concentration in craft beer to estimate which beverages may be hazardous for consumers [4].

BAs in samples of food and beverages are most commonly analysed using high-performance liquid chromatography (HPLC). However, the determination of BAs in the native state using methods based on reversed-phase liquid chromatography coupled with mass spectrometry may be problematic—hence the need for the derivatization step [8,9,10,13,14,15,16].

Numerous methods for the determination of BAs in beer samples have previously been reported, including HPLC-FLD, HPLC-UV, GC-MS, CE-UV, and CE-MS/MS [4,17,18,19,20]. However, to the best of our knowledge, none of these methods enable simultaneous determination of 17 BAs in a relatively short time (15 min). Additionally, the developed method based on MS detector working in MRM mode is highly sensitive (LOD and LOQ values are in the range: 0.00078–0.020 mg/L and 0.0023–0.059 mg/L, respectively).

In this work, the applicability of an analytical method based on derivatization and high-performance liquid chromatography-tandem mass spectrometry (HPLC-MS/MS) for simultaneous determination of a multitude of BAs in beer was tested. This method was developed and validated in our laboratory and described in detail in previous works [8,14]. Samples included commercially available beers, several of which were representative of styles in which LAB are involved in the fermentation process. The same method was used to monitor the concentration of several BAs throughout the fermentation process, including refermentation in the bottles. The total amount of BAs, a useful indicator of the risk associated with the consumption of the product resulting from ingestion of BAs, was also calculated.

## 2. Materials and Methods

### 2.1. Chemicals and Reagents

Biogenic amines standards methylamine hydrochloride 99% (MA), ethylamine hydrochloride 98%(EA), dimethylamine hydrochloride 99% (DMA), diethylamine hydrochloride 99% (DEA), propylamine hydrochloride 99% (PA), butylamine 99% (BA), 2-phenylethylamine hydrochloride 98% (PHA), tyramine hydrochloride 98% (TYR), tryptamine hydrochloride 99% (TRP), histamine dihydrochloride 99% (HIS), hexylamine 99%(HEA), isopentylamine 99% (isoPA), putrescine dihydrochloride 98% (PUT), cadaverine dihydrochloride 98% (CAD),spermidine trihydrochloride 99.5%(SPD), spermine tetrahydrochloride 99.5% (SPE), agmatine sulfate 98% (AGM) were obtained from Sigma Aldrich (St. Louis, MO, USA). The internal standard (IS) was 1.7-diamoheptane 98% (DAH) purchased from Sigma Aldrich (St. Louis, MO, USA). Formic acid (FA) was obtained from Merck (Darmstadt, Germany). Boric acid and sodium hydroxide were purchased from POCH (Gliwice, Poland). Acetonitrile (ACN) with LC-MS grade, ammonium formate and tosyl chloride (≥99%) were obtained from Sigma Aldrich (St. Louis, MO, USA). Nylon Captiva Econofilters (25 mm diameter, 0.2 µm pore size) were purchased from Agilent Technologies (Santa Clara, CA, USA). Ultrapure water was produced by the HLP5 system from Hydrolab (Wiślina, Poland).

### 2.2. Samples

Samples of commercially available beers listed in Table 1 were obtained from local distribution centres in Gdańsk, Poland. The beers were selected in order to reflect a wide range of variables which could possibly impact the concentration of BAs. Of the 13 different beers two were brewed by large breweries, while the others were brewed in craft and regional breweries; 4 were manufactured using bottom fermentation, 6 using top fermentation, and the remaining 3 were brewed with wild yeast. Six were purposefully inoculated with LAB during brewing in accordance with the style. The alcohol content of the finished beers ranged from 2.8% to 6.5%, and the concentration of the initial extract prior to fermentation ranged from 8% to 14.3%. All samples were well within the declared shelf-life and were stored in capped glass bottles in the dark until the analysis.

The ale for the determination of BAs during fermentation was home-brewed out of 5 kg of Pilsner malt and 0.25 kg of acidulated malt. The thermal program for mashing was as follows: 60 min at 66 °C, followed by 15 min at 72 °C and 5 min at 78 °C. The wort was hopped with 50 g of Galaxy^®^ hops (HPA, North Hobart, Australia) 10 min before the end of mashing and with 100 g of Kohatu^®^ (NZhops Ltd., Richmond, New Zealand) during fermentation; 11.5 g of Safale US-05 dry east were hydrated in 115 g of water at 35 °C. The end result was 32 L of wort with 10° Blg (100 g of sugars per 1 kg of wort, or 10% of extract by weight).

The wort was subsequently fermented in a dedicated polymeric fermenter at 15 °C. The temperature increased to 18 °C on day 9, and to 19 °C on day 11 of fermentation. On day 10 the concentration of sugars was 2%. Fermentation was terminated on day 19 with a cold crush down to approximately 0 °C. The ale was then poured into sterilised opaque glass bottles with an additional 2.5 g of sugar and 0.1 g of vitamin C for refermentation (in order to saturate the beverage with CO_2_ through the metabolic activity of yeast) and sealed with metal caps. Samples of 5 mL were collected approximately every 12 h during the stormy fermentation, and subsequently every 24 h until the end of fermentation using a disposable syringe through a spigot fitted with a silicon/PTFE septum mounted at the 10 L mark of the fermenter. The final sample was collected from the bottle 3 weeks after it was sealed. The usual care was taken to avoid contamination. The collected samples were frozen in Falcon tubes at −20 °C prior to the analysis.

**Figure 1:**
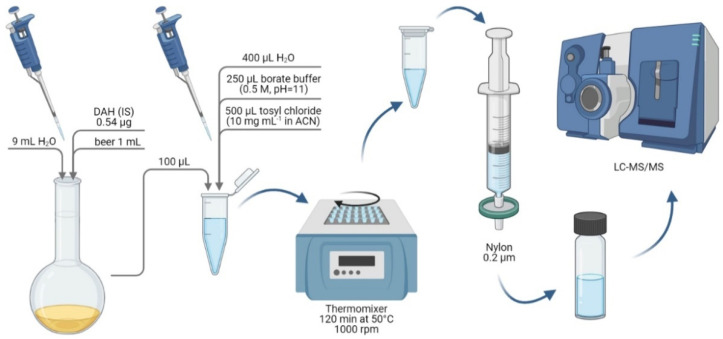
Schematic representation of the sample preparation stages for the determination of biogenic amines (BAs) in beer samples using liquid chromatography-tandem mass spectrometry (LC-MS/MS).

After thawing in the laboratory, the samples were subjected to an extraction procedure which was developed and described in detail in our previous papers [8,14]. Briefly, beer samples were degassed, diluted with water, subjected to derivatization with tosyl chloride, and filtered through a nylon filter. Finally, samples were injected into a chromatographic system. The sample preparation procedure is illustrated in Figure 1.

### 2.3. Instrumentation

All analyses were performed using the LCMS-8060 triple quadrupole mass spectrometer (Shimadzu, Kyoto, Japan) equipped with the ESI source operated in positive MRM ion mode following a previously validated method [8]. The chromatographic separation was performed using the UPLC Nexera X2 System (Shimadzu, Kyoto, Japan) equipped with the LC-30AD binary pump, DGU-20A5R degasser, CBM-20A controller, SIL-30AC autosampler, and CTO-20AC thermostated column oven. The separation of the derivatizated BAs was achieved using the Kinetex C8 (100 × 2.1 mm, 1.7 µm, Phenomenex, Torrance, CA, USA) column. The chromatographic separation conditions, MS/MS operation parameters (Table 2), and parameters of the monitored ion transitions (Supplementary Materials Table S1) are available in Supplementary Materials. Data acquisition and initial analysis were accomplished with LabSolutions 5.60 SP1 software. Further data processing and multivariate data analysis was performed using Orange v. 3.28 and Scikit-Learn v. 0.24 Python packages [21,22].

### 2.4. Standards and Calibration Solutions

The concentration of each of the BAs in the standards mix was 20 mg/L. The mix was prepared by adding 1 mL of each of the 17 stock solutions (500 mg/L) to a 25 mL volumetric flask and making up to the mark with ACN: 0.1 M HCl (3 + 7, *v*/*v*) mixture. Standard mix prepared in this way was used to construct calibration curves. Firstly, twelve calibration curves (0.001, 0.005, 0.025, 0.050, 0.10, 0.25, 0.50, 0.75, 1.00, 1.25, 1.50 and 1.75 mg/L) for each BA were prepared by diluting variable aliquots of the standard mix with ACN:0.1 M HCl (3 + 7, *v*/*v*) mixture. Next, in order to maintain linearity of the calibration curves, a six-point range was chosen individually for each BA from the 11 points. After this, 100 µL of each calibration solution was subjected to the same derivatization reaction as the beer samples. The concentration of IS (DAH) was kept at 0.050 mg/L in all calibration solutions. The individual ranges of the calibration curves for each BA and the remaining calibration parameters are listed in Supplementary Materials Table S2.

## 3. Results and Discussion

### 3.1. Determination of BAs in Samples of Commercially Available Beer

The results of the determination of BAs in samples of commercially available beer are listed in Table 3. In all types of beer, the most abundant BAs are AGM and PUT. However, in beers brewed with wild yeast (PIN, LMBC, GEU) the content of PUT was significantly higher than AGM (see Supplementary Materials Figure S1). This supports previously reported results [5]. In samples LMBC and APA2, PUT and AGM levels were approximately 20 mg/L and 50 mg/L, respectively, which might result in the enhancement of the toxicity of other BAs [23]. SPD was also present in all analysed samples, but its content was approximately 10 times lower in bottom fermentation and wild fermentation beers than in top fermentation beers (Supplementary Materials Figure S1). However, from a food safety point of view, the determined concentrations of SPD (<10 mg/L) can generally be considered to be of low risk for the consumer’s safety [4,11]. Levels of TYR in the 13 beer samples varied greatly. For example, in beer samples WZN and BWS2 contents of TYR were approximately 100–200 times higher than in in samples LGR3 and GEU. According to the literature, the acceptable concentration of BAs in the consumed product is up to 100 mg/kg or up to 100 mg/L [4]. However, in alcoholic beverages limits of BAs are significantly lower. Therefore, among BAs determined in the analysed samples, TYR seems to pose the highest health hazard, especially in the case of beer LGR3.

Controlling the level of HIS is very important due to the potential health risk to the consumer. In most cases the concentration of HIS ranged from 0.054 mg/L to 0.89 mg/L. However, in the case of samples LMBC and GEU its concentration exceeded the safety threshold in alcoholic beverages set at 2 mg/L [23]. A similar situation is observed for CAD: in all samples its level was lower than 1.14 mg/L. Only in the case of samples LMBC and GEU were higher concentrations observed. This corroborates previous reports on the high concentration of HIS and CAD in Belgian wild fermentation beers [5]. In other samples, the levels of BAs, especially those that may have a toxic effect on human health, such as TRP or SPE, were not significant. Moreover, in some cases (samples: LMBC, LGR2-4, BWS2, and GEU) at least one of the two BAs was not detected, and in one sample (sample PIN) both were not detected.

**Figure 2:**
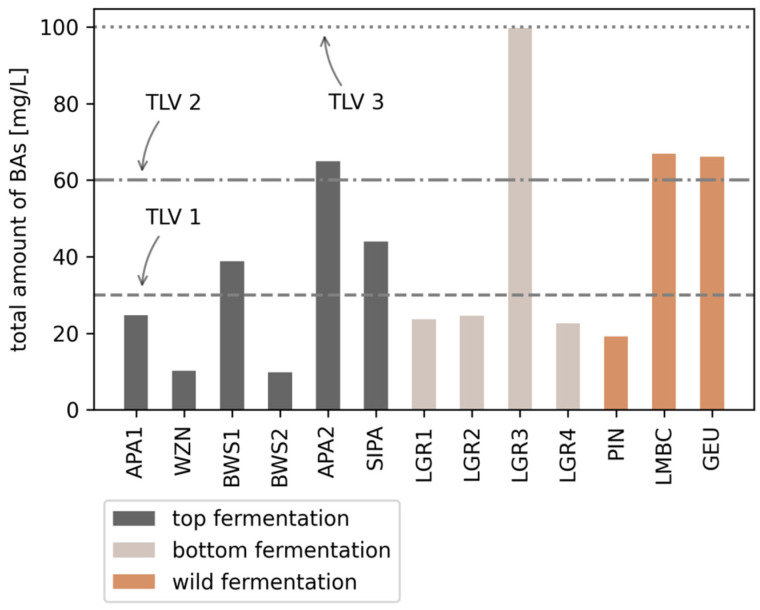
Total amount of BAs [mg/L] expressed as sum of 14 BAs (∑ MA, EA, DMA, PA, AGM, BUA, PUT, TRP, FEA, HIS, CAD, SPD, TYR, SPE) in 13 different beer samples. TLV 1 (threshold limit value)–risk for consumers with ineffective detoxification mechanism: 30 mg/L; TLV 2–risk for all consumers: 60 mg/L; TLV 3–general BAs threshold for foods: 100 mg/L).

In order to more easily assess the health hazard posed by the presence of BAs in foodstuffs their concentration is often expressed as an index (BAI–biogenic amines index) [24,25]. This is also true in the case of beers [5,26]. However, the most straightforward way to indicate the concentration of BAs in alcoholic beverages is to calculate their sum, or the total BAs concentration which was undertaken in this work. Here, the total amount of BAs is the sum of 14 BAs (Figure 2). DEA, isoPA and HEA were not included in the calculations due to the fact that they were not detected in any of the beer samples.

As shown in Figure 2, in the case of samples BWS1 and SIPA, the determined concentrations of BAs (30–50 mg/L) might be considered hazardous for some consumers–especially for those taking drugs which inhibit the activity of the detoxification system, people who have a genetically inefficient detoxification mechanism, or for people suffering from gastritis, irritable bowel syndrome, Crohn’s disease, and gastric and colon ulcers [4,27,28]. In samples LMBC, APA2, and GEU, the total content of BAs was above 60 mg/L which may be dangerous in combination with alcohol, even for healthy consumers due to the fact that alcohol weakens the detoxification [8,29]. In the case of one sample (sample LGR3), the total content of BAs was 100 mg/L, so these beverages can be considered hazardous for all consumers.

**Figure 3:**
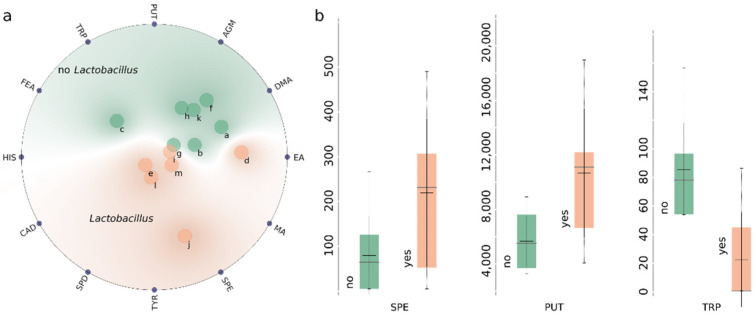
Untargeted (RadViz) analysis of commercial beer samples (**a**) and box plots for 3 most relevant BAs in terms of classification based on presence of lactic acid bacteria (**b**). Grey horizontal bars in box plots denote median values, and black horizontal bars denote average values. Labels in subplot (**a**): a-APA1, b-LGR1, c-WZN, d-PIN, e-LMBC, f-LGR2, g-LGR3, h-LGR4, i-BWS1, j-BWS2, k-APA2, l-GEU, m-SIPA.

Figure 3a shows a non-linear multi-dimensional 2d projection of the objects (samples) and variables (BAs) [30,31]. The points denoting beer samples that are closer to a set of BAs have higher values for these variables (after normalization) than for others. Beer samples can be relatively easily classified into those in which LAB were present during brewing and, conversely, those brewed without the use of LAB using such an unsupervised approach. A similar, but less definite classification can be achieved with respect to the presence of wild yeast (see Supplementary Materials Figure S1). The content of alcohol and initial concentration of sugars did not seem to significantly affect the concentration of BAs in the analysed beers. This is not surprising, since LAB, introduced either due to contamination or in accordance with the beer style, are one of the main sources of BAs in alcoholic beverages [15]. The lack of obvious correlation between the concentration of BAs and the characteristics of beer other than the presence of LAB also holds true for particular amines, as is the case e.g., with AGM which was present in some samples in relatively high concentration, but not consistently with the beer style.

The three most relevant variables (concentration of SPE, PUT, and TRP) in terms of supervised classification based on the presence of LAB, selected using the analysis of variance (ANOVA) are shown in Figure 3b.

### 3.2. Determination of BAs during Beer Fermentation

**Figure 4:**
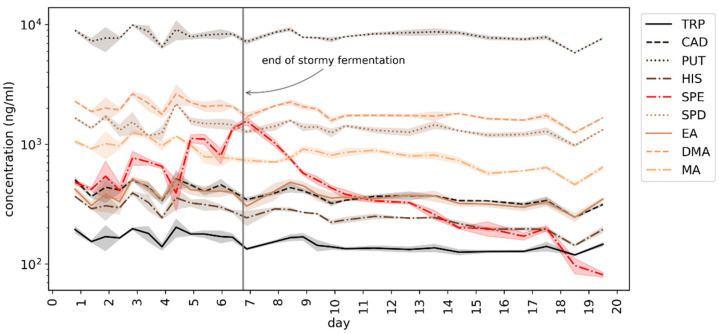
Changes in the concentration of BAs during fermentation of homebrewed ale. Note the logarithmic scale. Shaded areas denote sd (*n* = 3).

**Figure 5:**
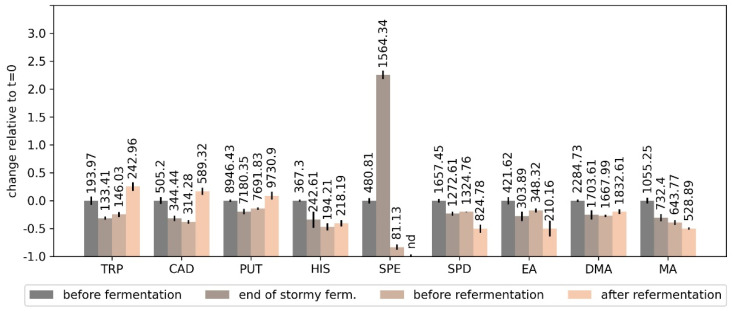
Change of the concentration of BAs during the production of homebrewed ale relative to the beginning of the fermentation process (0.0) at the beginning of fermentation, during bottling, and after refermentation in the bottles. Labels above the bars denote concentration in mg/L, and the bars themselves denote sd (*n* = 3).

The concentration of most of the monitored BAs did not change significantly throughout the fermentation process (Figure 4) which is consistent with previously reported results, as is the concentration level of the particular amines [32]. The exception was the concentration of SPE which increased until the end of stormy fermentation to then steadily decrease below the initial value (after mashing), and below the LOQ after refermentation in the bottles (see Figure 5). Such correlation with yeast activity could suggest a link with the metabolic activity of yeast. SPE is the final product of biosynthetic pathways for polyamines. While it was previously thought that brewer’s yeast are unable to form BAs [33], *Saccharomyces cerevisiae* do contain the SPE4 gene which codes for SPE synthase, allowing for synthesizing SPE from SPD [34]. However, the concentration of BAs in beer can be affected by a multitude of factors, including the raw materials, microbiological contaminants, and yeast strains. It was previously indicated that the decrease in the concentration of SPE in the final product compared to its concentration in raw materials can be the result of its transformation into 1,3-aminopropylpyrroline together with 1,3-diaminopropane by amino oxidases present in barley [32]. However, this would most likely take place during mashing, not during fermentation.

## 4. Conclusions

A method for simultaneous determination of a multitude of BAs in beer was successfully used to analyse several samples of commercially available beers, focusing on the dichotomy of styles which can and ought not to involve lactic fermentation.

Multivariate statistical analysis has revealed differences between the two subsets, likely due to the fact that lactic acid bacteria are one of the sources of BAs in alcoholic beverages.

In all tested samples, the highest level was noted for PUT and AGM, while in beers brewed with wild yeast (PIN, LMBC, GEU) the content of PUT was significantly higher than AGM. In order to estimate the risk associated with beer consumption, the total content of BAs in the beer samples was calculated. Bearing in mind that the generally safe limit value of the total content of BAs is 100 mg/L (while in alcoholic beverages this threshold is much lower), it was observed that 4 out of 13 beers (sample LGR3, LMBC, GEU, APA2) in which the total BA content exceeded 60 mg/L may pose a risk even to a healthy consumer. Two samples out of 13 (sample BWS1 and SIPA) in which the total BAs content ranged between 30–50 mg/L can be considered dangerous for some—especially those taking drugs that inhibit the detoxification system, people who have a genetically ineffective detoxification mechanism, or people suffering from gastritis, irritable bowel syndrome, Crohn’s disease, and gastric and colon ulcers. In other cases, the total amount of BAs was not significant for human health (<30 mg/L). Generally, the total content of BAs in beers brewed by large breweries (LGR2 and LGR4) was significantly lower compared to beers brewed in craft and regional breweries. Based on the results obtained it might be concluded that it is necessary to monitor the total level of BAs in beer. Information on the content of BAs may be particularly useful for people who take medication or have an inefficient detoxification mechanism. It was also demonstrated that the procedure can be used for monitoring the changes in concentration of particular BAs during the fermentation stage of beer manufacturing, granting a valuable insight into the process. In particular, it was observed that the concentration of SPE in the wort increased until the end of the stormy fermentation stage, to then drop below the initial concentration at the end of fermentation, and below the LOQ (0.015 mg/L) after refermentation.

## Figures and Tables

**Table 1:** Tested samples of commercially available beers.

Label	Style	Alcohol (%)	Extract (%)	Yeast	Lactobacillus	Other
PIN	wild sour ale pinot noir barrel aged	6.5	14.3	Wild	Yes	barrel aged
LMBC	lambic	4.5	12	Wild	Yes	addition of Candi sugar
GEU	geuze	5	12	Wild	Yes	
LGR1	lager	5	12	Bottom	No	
LGR2	lager	5.2	11.5	Bottom	No	
LGR3	lager	5.4	12.2	Bottom	No	
LGR4	lager	5	12	Bottom	No	
WZN	weizen	5	12.5	Top	No	wheat
BWS1	Berliner weisse	5	11.7	Top	Yes	
BWS2	Berliner weisse	2.8	8	Top	Yes	
APA1	pale ale	5	12	Top	No	
APA2	pale ale	3	12.5	Top	No	
SIPA	sour india pale ale (IPA)	6	16	Top	Yes	

**Table 2:** The chromatographic separation conditions and MS/MS operation parameters.

Chromatographic Separation Condition	
Column	Kinetex C8 (100 × 2.1 mm, 1.7 µm)
Flow rate (mL/min)	0.6
Temperature of thermostat (°C)	40 °C
Injection volume	1 µL
Analysis time	15 min
Mobile phase	A: H_2_O 0.1% *v*/*v* FA
	B: ACN 0.1% *v*/*v* FA
Gradient elution	0 → 5 min 25–40% B
	5 → 10 min 40–80% B
	10 → 15 min 25% B
MS/MS Operation Parameters	
Source temperature (°C)	300
Ion spray voltage (kV)	4
Nebulizing gas flow (L/min)	3
Heating gas flow (L/min)	10
Drying gas flow (L/min)	10
Temperature of the desolvation line (°C)	250
Heat block (°C)	400

**Table 3:** Results of the determination of BAs in samples of commercially available beer (mg/L) ± sd (*n* = 3).

Beer	MA	EA	DMA	PA	AGM	BUA	PUT	TRP	FEA	HIS	CAD	SPD	TYR	SPE	Sum of BAs
PIN ^a^	0.397 ± 0.016	0.793 ± 0.018	0.3829 ± 0.0091	0.462 ± 0.078	N/A	N/A	6.63 ± 0.49	N/A	0.5542 ± 0.0051	0.333 ± 0.0042	0.2594 ± 0.0052	0.1929 ± 0.0034	9.72 ± 0.13	N/A	19.2
LMBC ^a^	1.874 ± 0.289	0.359 ± 0.072	0.54 ± 0.12	N/A	1.34 ± 0.21	2.958 ± 0.020	19.0 ± 2.4	N/A	0.145 ± 0.040	4.960 ± 0.067	3.646 ± 0.053	0.2823 ± 0.0040	31.71 ± 0.42	0.0522 ± 0.0023	66.8
GEU ^a^	0.905 ± 0.050	0.0566 ±0.0097	0.932 ± 0.052	N/A	5.08 ± 0.29	N/A	11.91 ± 0.36	N/A	0.1091 ± 0.0066	2.20 ± 0.22	3.12 ± 0.13	0.475 ± 0.015	41.2 ± 1.4	0.172 ± 0.013	66.1
LGR1 ^b^	0.333 ± 0.050	0.189 ± 0.032	0.67 ± 0.12	N/A	14.23 ± 0.16	N/A	5.51 ± 0.70	76.6 ± 0.0034	N/A	0.06046 ± 0.00056	0.2778 ± 0.0036	1.3248 ± 0.0087	0.758 ± 0.015	0.2662 ± 0.0081	23.7
LGR2 ^b^	0.416 ± 0.038	0.299 ± 0.015	0.732 ± 0.016	N/A	16.160 ± 0.017	N/A	4.903 ± 0.036	0.0782 ± 0.0014	N/A	0.8887 ± 0.0021	0.4006 ± 0.0027	0.1538 ± 0.0021	1.37 ± 0.031	N/A	24.6
LGR3 ^b^	0.505 ± 0.066	0.647 ± 0.021	0.6232 ± 0.0071	N/A	18.21 ± 0.14	N/A	7.616 ± 0.036	0.1567 ± 0.0042	0.156 ± 0.016	0.1919 ± 0.0057	0.989 ± 0.033	0.4319 ± 0.0097	70.24 ± 0.72	N/A	99.8
LGR4 ^b^	0.449 ± 0.044	0.268 ± 0.011	0.703 ± 0.016	N/A	12.33 ± 0.31	N/A	5.79 ± 0.62	0.0967 ± 0.0015	N/A	0.1288 ± 0.0016	1.140 ± 0.018	0.0536 ± 0.0029	1.617 ± 0.069	N/A	22.6
WZN ^c^	0.204 ± 0.025	0.090 ± 0.015	0.3434 ± 0.0093	N/A	3.814 ± 0.026	N/A	3.284 ± 0.053	0.54051 ± 0.00052	1.037 ± 0.022	0.1213 ± 0.0032	0.3158 ± 0.0016	0.495 ± 0.014	0.36968 ± 0.00069	0.1262 ± 0.0041	10.3
BWS1 ^c^	0.168 ± 0.015	0.0456 ± 0.0058	0.499 ± 0.021	N/A	22.66 ± 0.45	N/A	10.29 ± 0.24	0.0451 ± 0.0015	N/A	0.394 ± 0.016	0.5349 ± 0.0096	2.822 ± 0.065	1.071 ± 0.026	0.307 ± 0.030	38.8
BWS2 ^c^	N/A	N/A	0.127 ± 0.011	N/A	3.596 ± 0.088	N/A	4.050 ± 0.071	N/A	N/A	0.213 ± 0.016	0.2701 ± 0.0072	0.913 ± 0.012	0.3978 ± 0.0011	0.2908 ± 0.0077	9.9
APA1 ^c^	0.580 ± 0.035	0.237 ± 0.043	1.23 ± 0.10	N/A	16.54 ± 0.12	N/A	3.69 ± 0.17	0.05421 ± 0.00078	N/A	0.0540 ± 0.0010	0.2680 ± 0.0089	0.927 ± 0.014	1.011 ± 0.014	0.8331 ± 0.0098	24.7
APA2 ^c^	0.755 ± 0.013	0.1221 ± 0.0066	1.544 ± 0.0091	N/A	48.60 ± 0.44	N/A	8.91 ± 0.21	0.0832 ± 0.0024	0.5519 ± 0.0015	0.306 ± 0.025	0.849 ± 0.032	1.808 ± 0.041	1.871 ± 0.029	0.0647 ± 0.0025	65.0
SIPA ^c^	0.644 ± 0.041	0.1162 ± 0.0025	0.866 ± 0.021	N/A	21.27 ± 1.00	N/A	12.20 ± 0.58	0.0864 ± 0.0085	0.5252 ± 0.0062	0.344 ± 0.016	0.6870 ± 0.0028	5.112 ± 0.049	2.104 ± 0.062	0.4907 ± 0.0069	44.0

^a^ Wild yeast; ^b^ bottom fermentation yeast; ^c^ top fermentation yeast; N/A—below LOQ.

## Data Availability

The data presented in this study are available in the Supplementary Materials.

## Supplementary Materials

The following are available online at https://www.mdpi.com/article/10.3390/foods10122902/s1, Table S1: Parameters of the monitored ion transitions, Table S2: The individual ranges of the calibration curves for each BA and the remaining calibration parameters, Figure S1: Box plots for selected BAs grouped by the type of fermentation. Grey horizontal bars denote median values, and black horizontal bars denote average values.
