# Supplementary material for: Occurrence and Levels of Biogenic Amines in Beers Produced by Different Methods
Source: Foods. 2021 Nov 23;10(12):2902. doi: 10.3390/foods10122902 (PMC8700637; doi:10.3390/foods10122902)
Supplement: Supplementary file 1 [file foods-10-02902-s001.zip › foods-1443431-supplementary.pdf]

Article

Occurrence and Levels of Biogenic Amines in Beers Produced by Different Methods

Katarzyna Nalazek-Rudnicka, Wojciech Wojnowski and Andrzej Wasik *

Table S1. Parameters of the monitored ion transitions.

Compounds	Transitions	Q1	CE	Q3
EA	200→155	-14	-11	-10
	200→91	-14	-23	-16
DMA	200→155	-13	-14	-27
	200→91	-10	-24	-19
PA	214→155	-15	-14	-27
	214→91	-16	-24	-15
BA	228→155	-18	-13	-15
	228→91	-15	-27	-17
DEA	228→155	-16	-14	-15
	228→91	-11	-25	-16
isoPA	242→155	-22	-13	-22
	242→91	-24	-29	-16
FEA	276→105	-13	-15	-21
	276→91	-23	-42	-15
HEA	256→155	-16	-15	-15
	256→91	-10	-24	-16
HIS	420→155	-20	-25	-20
	420→91	-20	-35	-20
TRP	315→144	-20	-13	-14
	315→117	-11	-41	-22
TYR	446→275	-12	-15	-12
	446→155	-12	-23	-14
PUT	397→226	-20	-18	-14
	397→155	-18	-25	-15
CAD	411→240	-15	-17	-15
	411→184	-15	-20	-18
AGM	439→155	-16	-29	-15
	439→91	-16	-48	-15
DAH	439→155	-12	-27	-15
	439→91	-10	-53	-17
SPD	608→383	-22	-18	-18
	608→226	-30	-25	-14
SPE	819→212	-22	-31	-13
	819→281	-22	-33	-18
MA	186→155	-22	-13	-23
	186→91	-13	-22	-17

Table S2. The individual ranges of the calibration curves for each BA and the remaining calibration parameters.

BAs	Range [mg/L]	Calibration curve equations	S_a	S_b	LOD [mg/L]	LOQ [mg/L]
MA	0.005-0.75	y = 0.000528x + 0.010917	0.00000947	0.000663	0.0041	0.012

EA	0.005-0.75	y = 0.001531x + 0.00785	0.0000133	0.000933	0.0020	0.0060
DMA	0.005-1.00	y = 0.005957x + 0.10081	0.000113	0.00875	0.0049	0.015
PA	0.025-0.25	y = 0.001261x + 0.05182	0.0000391	0.00382	0.010	0.030
AGM	0.050-1.75	y = 0.001306x - 0.00663	0.0000211	0.00731	0.018	0.055
BUA	0.005-0.25	y = 0.001044x + 0.10017	0.0000399	0.00158	0.0050	0.015
DEA	0.005-0.25	y = 0.004492x + 0.06163	0.000151	0.00596	0.0044	0.013
PUT	0.050-1.75	y = 0.007863x - 0.1033	0.000136	0.0472	0.020	0.059
TRP	0.005-0.75	y = 0.003458x + 0.015074	0.0000117	0.000818	0.00078	0.0023
FEA	0.005-0.75	y = 0.001899x + 0.00476	0.000036	0.00252	0.0044	0.013
isoPA	0.005-0.50	y = 0.001107x + 0.03574	0.000025	0.00145	0.0043	0.013
HIS	0.005-0.75	y = 0.015561x + 0.0659	0.000328	0.0231	0.0049	0.015
CAD	0.005-0.75	y = 0.004062x + 0.01287	0.000076	0.00531	0.0043	0.013
HEA	0.005-0.25	y = 0.001324x + 0.04071	0.0000501	0.00198	0.0049	0.015
SPD	0.005-0.75	y = 0.009759x + 0.0156	0.000209	0.0147	0.0050	0.015
TYR	0.005-0.75	y = 0.0028411x + 0.00807	0.0000201	0.00143	0.0017	0.0050
SPE	0.005-0.75	y = 0.003351x + 0.01641	0.0000713	0.00499	0.0049	0.015

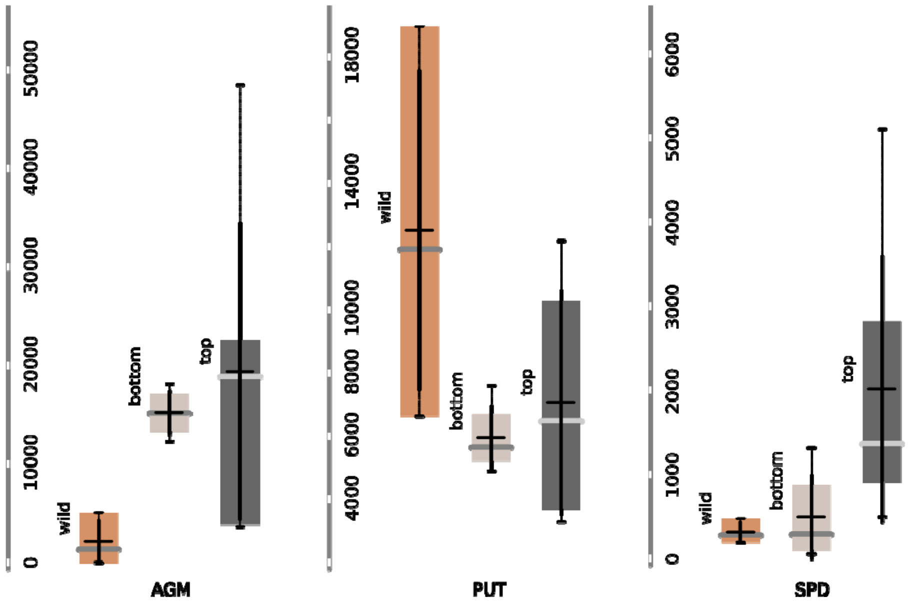

Figure S1. Box plots for selected BAs grouped by the type of fermentation. Grey horizontal bars denote median values, and black horizontal bars denote average values.
